# Screening uptake rates and the clinical and cost effectiveness of screening for gestational diabetes mellitus in primary versus secondary care: study protocol for a randomised controlled trial

**DOI:** 10.1186/1745-6215-15-27

**Published:** 2014-01-17

**Authors:** Angela O’Dea, Jennifer J Infanti, Paddy Gillespie, Olga Tummon, Samuel Fanous, Liam G Glynn, Brian E McGuire, John Newell, Fidelma P Dunne

**Affiliations:** 1 School of Medicine, Clinical Sciences Institute, National University of Ireland Galway, Galway, Ireland; 2 J.E. Cairnes School of Business & Economics, Cairnes Building, National University of Ireland Galway, Galway, Ireland; 3 Discipline of General Practice, School of Medicine, 1 Distillery Road, National University of Ireland Galway, Galway, Ireland; 4 School of Psychology, National University of Ireland Galway, University Road, Galway, Ireland; 5 HRB Clinical Research Facility Galway, National University of Ireland Galway, University Road, Galway, Ireland

**Keywords:** Gestational diabetes mellitus, Screening, Primary care, Secondary care, Randomised controlled trial

## Abstract

**Background:**

The risks associated with gestational diabetes mellitus (GDM) are well recognized, and there is increasing evidence to support treatment of the condition. However, clear guidance on the ideal approach to screening for GDM is lacking. Professional groups continue to debate whether selective screening (based on risk factors) or universal screening is the most appropriate approach. Additionally, there is ongoing debate about what levels of glucose abnormalities during pregnancy respond best to treatment and which maternal and neonatal outcomes benefit most from treatment. Furthermore, the implications of possible screening options on health care costs are not well established. In response to this uncertainty there have been repeated calls for well-designed, randomised trials to determine the efficacy of screening, diagnosis, and management plans for GDM. We describe a randomised controlled trial to investigate screening uptake rates and the clinical and cost effectiveness of screening in primary versus secondary care settings.

**Methods/Design:**

This will be an unblinded, two-group, parallel randomised controlled trial (RCT). The target population includes 784 women presenting for their first antenatal visit at 12 to 18 weeks gestation at two hospitals in the west of Ireland: Galway University Hospital and Mayo General Hospital. Participants will be offered universal screening for GDM at 24 to 28 weeks gestation in either primary care (n = 392) or secondary care (n = 392) locations. The primary outcome variable is the uptake rate of screening. Secondary outcomes include indicators of clinical effectiveness of screening at each screening site (primary and secondary) including gestational week at time of screening, time to access antenatal diabetes services for women diagnosed with GDM, and pregnancy and neonatal outcomes for women with GDM. In addition, parallel economic and qualitative evaluations will be conducted. The trial will cover the period from the woman’s first hospital antenatal visit at 12 to 18 weeks gestation, until the completion of the pregnancy.

**Trial registration:**

Current Controlled Trials: ISRCTN02232125

## Background

Gestational diabetes mellitus (GDM) has been defined by the World Health Organization (WHO) as hyperglycaemia of variable severity with onset or first recognition during pregnancy [1]. The risks associated with gestational diabetes mellitus are well recognized. Pregnancies complicated by GDM are associated with increased incidence of both adverse maternal outcomes (pregnancy-induced hypertension, polyhydramnios and caesarean section) and neonatal outcomes (prematurity, large for gestational age, neonatal unit admission, neonatal hypoglycaemia and respiratory distress) [2,3]. Later in life, both the mother and child are at increased risk of hospital admission, obesity, type 2 diabetes and heart disease [2,4-7]. Fortunately, these complications seem to be lessened by better detection and management of the condition [8,9].

There is a lack of robust evidence relating to the prevalence of GDM internationally [10,11]. This is due in large part to the use of a wide range of definitions and diagnostic criteria [12-14], as well as variations in prevalence across regions and ethnic groups [10,15].

Nonetheless, there is evidence that GDM affects significant numbers of pregnancies each year and that the prevalence of GDM has been increasing rapidly in recent years [16]. In Ireland, a large-scale research study funded by the Health Research Board and conducted by the Atlantic Diabetes in Pregnancy (Atlantic DIP) consortium, found that 12.4% of pregnancies in Ireland are complicated by GDM [17] according to the IADPSG (International Association of Diabetes and Pregnancy Study Group) diagnostic criteria [12].

Despite the increasing prevalence of the disease and the adverse consequences associated with it, there is still significant uncertainty about the screening, diagnosis and management of GDM [10,15].

In terms of screening, for example, professional groups disagree on whether to recommend universal or selective screening (based on risk factors) [12,18,19]. While some studies have found that universal screening for GDM is superior to risk factor-based screening in terms of number of cases detected, time to diagnosis and treatment, and improved pregnancy outcomes [20,21], others have found no such effects. For example, a review of four trials comparing universal and selective screening, found that there is little high-quality evidence on the effects of screening for GDM on health outcomes for mothers and their babies. The paper concluded that further research is required to see which recommendations for screening practices for GDM are most appropriate [22].

Additionally, the site of screening is likely to be important, both in terms of the location of the screen (regional versus local) and in terms of the provider of the screening service (GP versus hospital). For example The Atlantic DIP study, which offered universal screening to all pregnant women, recorded an uptake rate of only 44% [17]. Follow-up analysis to explain this low screening uptake rate revealed that travel distance to the hospital screening site is an important factor in determining uptake rates [23]. Specifically, the probability of attending for screening is 15% lower for a patient who lives 50 kilometers from a screening centre, compared to an otherwise similar patient who lives closer to the hospital. The corresponding reduction in the probability of attending for an individual residing 100 kilometers away is 30%. This suggests that the availability of GDM screening in local (primary care) settings could result in higher uptake rates; however, this has not been empirically tested to date.

It is possible that service provider differences may impact on uptake rates too. Given that the oral glucose tolerance test (OGTT) screen involves three blood draws and takes in excess of two hours to complete, it is conceivable that factors such as wait time, space and comfort in the waiting area, and perceived skill of the phlebotomist/nurse/GP, may influence the decision to attend. However, to date, no evidence exists on this issue. Additionally, evidence is needed in order to determine if the site of GDM screening will impact health outcomes; It is possible that there may be differences between primary and secondary care in terms of timeliness of screening, access to results, communication of results to patients, or access to GDM treatment for those with positive test results.

There is also a lack of consensus surrounding which pregnancy outcomes can be influenced by GDM management. Earlier published work found little evidence for an effect of treatment on various perinatal outcomes [24,25]. A 2003 Cochrane Collaboration systematic review also concluded that 'there are insufficient data for any reliable conclusions about the effects of treatments for impaired glucose tolerance on perinatal outcomes’ [26]. However, more recent evidence has shown a beneficial effect of treatment. For example, in a RCT comparing GDM treatment versus routine care for mildly diabetic patients (that is, those not having overt diabetes), Crowther [8] found that treatment for GDM reduced the rate of serious perinatal outcomes (death, shoulder dystocia, bone fracture, and nerve palsy) from 4% to 1%. Rates of caesarean section delivery were similar in the two groups. On the other hand, another study [27] showed no significant difference in a combined endpoint analysis between treated and untreated women, although treatment did lower the risks of fetal overgrowth, shoulder dystocia, caesarean delivery and pre-eclampsia. Recent meta-analyses have shown treatment effects for some perinatal complications - for example, shoulder dystocia, large for gestational age (LGA) and macrosomia in the infant, and pre-eclampsia in the mother [28-30]. Thus, while the evidence is mounting for the benefits of treatment there is still uncertainty about which outcomes can be influenced and, moreover, what degree of carbohydrate intolerance will benefit most from treatment [28,31]. In response to this uncertainty there have been repeated calls for well-designed, randomised trials to determine the efficacy of screening, diagnosis, and management of GDM.

### Screening in Ireland

In Ireland, although guidelines exist, there is no nationally implemented screening policy for GDM. However, the Atlantic DIP network^a^[1] advocates a universal screening approach for all pregnant women [17]. Part of the rationale for this policy is that research has shown that implementation of selective screening would decrease the number of screens by only 10% while adding significantly to the complexity of the screening process [32]. Currently in Ireland, GDM screening is only offered at secondary care sites. Limiting screening to hospital sites can result in geographical inequalities in access to screening. Additionally, since screening for GDM facilitates the identification of women who are at risk of developing type 2 diabetes in the future and who should undergo long-term health surveillance and preventative measures, women residing in more remote locations are more disadvantaged in terms of both their short- and long-term health.

Thus, it is important to understand if offering a screening programme in primary care would improve uptake rates, access to treatment and pregnancy outcomes. Finding answers to these questions could have important implications for the planning and provision of future GDM screening services nationally. The challenge to the health service is to provide a screening programme that accurately identifies the population at risk and is able to ensure that the uptake rate in the population at risk is on par with international norms for screening programmes. In order to do this, evidence is required on the clinical and cost effectiveness of alternative screening models that could be introduced. A primary versus secondary care based universal screening programme for GDM has not been previously explored or evaluated.

In this paper, we detail an RCT to compare the relative clinical and cost effectiveness of a primary versus secondary care based universal screening programme for GDM. The primary outcome variable is screening uptake rate at each site. Secondary outcomes include gestational age at time of screen, timing of access to antenatal diabetes care services for women with GDM, and pregnancy outcomes for women with GDM. In parallel, we will investigate the cost implications of primary versus secondary care screening. We will also conduct a qualitative analysis of user and stakeholder attitudes, preferences, barriers and facilitators of screening at each site.

#### Objectives

The aim of the trial is to assess the uptake, clinical and cost effectiveness of universal screening for GDM in primary care versus secondary care locations.

### Hypothesis

The formal null hypothesis to be tested in this study is that there is no difference in uptake of screening for GDM amongst women randomised to primary care screening compared to women randomised to secondary care screening.

## Methods/Design

### Unblinded, parallel group, randomised controlled trial

A randomised controlled trial will be conducted to compare the uptake, clinical effectiveness and cost effectiveness of universal screening for GDM in primary versus secondary care. The CONSORT guidelines [33] for the conduct of RCTs will be employed in the design and analysis of the trial. The trial is registered with the International Standard Randomised Controlled Trial Number, ISRCTN02232125.

### Interventions

All women who consent to participate in the study will be invited for a 2-hour, 75 g, oral glucose tolerance test (OGTT) at between 24 to 28 weeks gestation. Participants will be randomised into either the primary care screening group or the secondary care screening group. The scheduling process differs slightly between groups. In the primary care group, the GP is requested to schedule the OGTT screening appointment with the woman; in the secondary care group researchers schedule the screening appointment during the recruitment phone calls. In neither group is the responsibility for scheduling the appointment left to the woman.

Primary care screening group: All women randomised to the primary care screening group will be offered a screening appointment for GDM at their local general practitioner (GP) clinic. Following randomisation, a study information leaflet and the materials required to perform an OGTT will be sent to the woman’s GP by the research team. The GP is requested to contact the woman in order to schedule the OGTT appointment at 24 to 28 weeks gestation. As per usual care, the GP will send the test samples to the local laboratory for analysis. Following analysis, the laboratory will send the test results directly to the GP and the GP will inform the woman of her result. For positive test results, the GP will be directed to contact the local maternity unit to make an appointment for the woman to be seen at the diabetes antenatal service.

Secondary care screening group: All women randomised to the secondary care screening group will be offered a screening appointment at the hospital site. Following randomisation, the study researcher will schedule the appointment for the OGTT screen and send the list of OGTT appointments to the maternity unit, for inclusion in their OGTT appointment diary. As per usual care, the test samples will be sent to the local laboratory for analysis. Following analysis, the laboratory will send the test results directly to the consulting obstetrician, who in turn informs the woman of her result. For positive test results, an appointment will be made by the midwife for the woman to attend the antenatal diabetes service.

#### Setting

This study will run concurrently at two sites: Galway University Hospital (GUH) and Mayo General Hospital (MGH) and their associated network of general practitioner (GP) clinics and primary care teams. GUH is the lead Atlantic DIP network site with approximately 4,000 deliveries per year. GUH serves both urban and rural populations, including Galway city, the third largest city in Ireland. MGH serves a rural population and has approximately 2,300 deliveries per year. Thus, GUH and MGH patients can be considered as broadly representative of the general population of Ireland. Within this geographical region is a network of general practitioners (Western Research and Educational Network: WestREN) who are interested in pursuing research in the community in collaboration with colleagues in secondary care. WestREN includes more than 80 general practices and more than 400,000 patients along the western seaboard of Ireland. Recently published research confirms that the WestREN practices and patients are representative of Ireland generally [34].

In advance of the commencement of the trial, all GPs in the Galway and Mayo region will be sent a trial information leaflet and asked to formally opt-out if they do not wish to participate. Additionally, GPs will be instructed to inform the study team if they are unable to perform the OGTT test, in such circumstances the participant will be offered a screen in secondary care.

#### Participants

The recruitment population is pregnant women presenting for their first antenatal visit at GUH and MGH at 12 to 18 weeks gestation. Participation in the study ends on completion of the pregnancy. All expectant mothers in Ireland are entitled to the Maternity and Infant Care Scheme, which consists of a shared programme of care provided by GPs in primary care and obstetricians in the hospital maternity units, or secondary care. The care scheme provides for an initial examination with the GP before 12 weeks gestation, followed by a hospital appointment and scan at 12 to 18 weeks gestation, followed by 6 further examinations alternated between the GP clinic and the maternity unit over the course of the pregnancy. Screening for GDM is embedded in hospital practice and supported by protocols.

#### Eligibility criteria

•Inclusion criteria: Women presenting with a pregnancy at GUH and MGH during the recruitment phase will be included.

•Exclusion criteria: Women with established diabetes or diagnosed GDM will be excluded.

#### Recruitment

**Figure 1:**
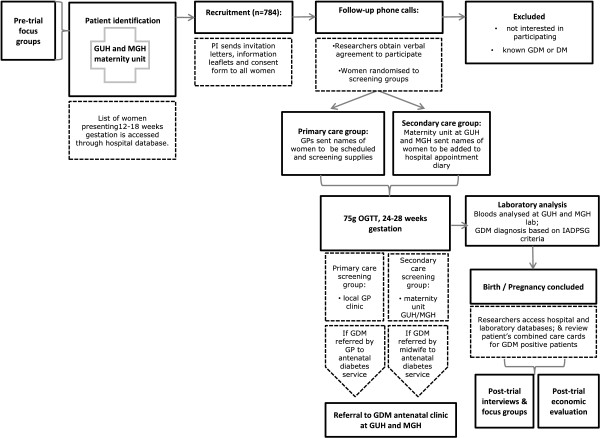
Participant flow through recruitment process.

Recruitment for the trial will be initiated at the woman’s first hospital visit at 12 to 18 weeks gestation. A list of all women with a confirmed pregnancy presenting at the obstetrics outpatient clinics at GUH and MGH will be obtained from the hospital databases by the principal investigator, who is a member of the clinical care team at both sites. Each woman will be sent an invitation letter, participant information leaflet and participant consent form in the post. The information will be followed up with a phone call to each woman to answer questions and obtain verbal consent before randomising those who consent to the primary care and secondary care screening groups. The baseline characteristic questionnaire will be administered at this time. The phone calls will be conducted by members of the research team. Each consenting woman will be asked to return the written consent form to the study office. For some of the participants (that is, those who meet the risk factor criteria for selective GDM screening), an appointment for an OGTT may have already been scheduled at their 12 to 18 week hospital visit. In such cases, researchers will continue the randomisation process as usual and inform the clinic if a change is required to the screening schedule. Those who were not selected for screening at the 12 to 18 week clinic but who consent to the study and are randomised into the secondary care screening group will be added to the hospital’s screening schedule. Outcome data will not be collected from participants who discontinue. The flow chart in Figure 1 represents the movement of a participant through the stages of recruitment.

#### Sample size and power calculation

On the basis of a comparison of two proportions a sample of 784 is needed to have 80% power, at the 5% significance level, to detect a ≥10% change in screening uptake. To achieve the required sample size, 1,513 patients will be invited to participate in the RCT, as 74% recruitment and 30% patient attrition (seen in Atlantic DIP) is anticipated. Recruitment will continue until target sample size is reached.

#### Randomisation

The randomisation process in this RCT will use random permuted blocks to ensure similar numbers of participants in each intervention arm throughout the trial and equal numbers in each arm by the end of the study. Blocks of varying length will be used to reduce the predictability of the allocation sequence. In advance of participant recruitment, an independent researcher will be responsible for generating the allocation sequence using the NQuery statistical software program (version 2.0). (Statistical Solutions, Cork, Ireland; http://www.statistical-solutions-software.com) Sealed envelopes will be used to assign participants to intervention groups. Thus, the allocation sequence will be concealed from all study researchers until the interventions are assigned.

#### Laboratory analysis

All test samples will be analysed at the local hospital laboratory. For those women presenting at GUH, analysis will take place at the GUH laboratory; for those women presenting at MGH, analysis will take place at the MGH Laboratory. Each test sample will have a study-specific label to enable identification. Laboratory samples for plasma glucose were analysed using the hexokinase method (Roche Modular P Analytics Chemistry Systems). GDM will be defined according to the new International Association of Diabetes and Pregnancy Study Groups (IADPSG) criteria [35]. We will apply the cut-off values for a diagnosis of GDM as follows: fasting, 1 hour or 2 hour glucose values of ≥5.1 mmol/l (92 mg/dl), 10 mmol/l (180 mg/dl) or 8.5 mmol/l (153 mg/dl), respectively.

#### GDM diagnosis and treatment, antenatal, delivery and neonatal care

All women with positive test results will be offered GDM treatment. This will involve lifestyle intervention, blood glucose self-monitoring and, if required, insulin. In terms of lifestyle intervention, all women will be offered consultations with a dietician and a diabetes nurse specialist. In terms of blood glucose self-monitoring, all women will be educated and instructed to self-monitor over the remainder of their pregnancy. Women who do not respond to lifestyle intervention after two weeks will be prescribed insulin for the remainder of the pregnancy. Antenatal care for all women with GDM will be provided by specialists in diabetes and obstetrics in secondary care and by the woman’s GP. All deliveries will take place at the local hospital.

#### Data collection

Data collection has two components: baseline characteristic data and clinical outcome data. These are described below.

**Table 1:** Baseline characteristic data

Clinical risk factors	Age
	Ethnicity
	Body mass index
	Parity
	Personal and family history of diabetes
	Smoking status
	Hypertension
	Previous pregnancy ending in perinatal death
	Current medications
	Expected date of delivery
Socioeconomic status	Medical card status^b^
	Private health insurance status
	Employment status
	Education status
	Marital status
	Distance and journey time from hospital and GP settings
	Mode of transport

#### Baseline characteristic data

In order to compare participants in both screening arms, clinical risk factor data and socioeconomic status data will be collected during the recruitment phase, using the baseline characteristic questionnaire (Table 1).

#### Clinical outcome data

Data on a range of clinical outcomes will be collected to explore the clinical effectiveness of the alternative screening programmes. Two sources of data are used:

•Hospital and laboratory database reviews: Data will be extracted from the hospital and laboratory databases on test results, processes of care and pregnancy outcomes.

•Chart searches: Antenatal care documents (combined care cards) will be reviewed at the end of the study to capture processes of care for GDM women, over the course of the pregnancy.

### Outcomes

#### Primary outcome: uptake of screening

The primary outcome in the RCT is uptake of screening in the primary care and secondary care settings. First, the number of screening offers accepted will be assessed by comparing the number of screens offered (via the obstetrics outpatient clinic list) with the number of offers accepted (via the randomisation schedule). Second the uptake rate will be determined by comparing the number of women randomised to each setting to the number of OGTTs performed in that setting (via the GUH and MGH laboratory records).

#### Secondary outcome 1: GDM prevalence

The prevalence of GDM in primary and secondary care settings is a secondary outcome in the RCT. Analysis of the laboratory database will enable the identification of GDM prevalence in both screening groups.

#### Secondary outcome 2: timing of screening

The timing of screening in primary and secondary care settings is a secondary outcome in the RCT. Analysis of the laboratory database will enable a determination of gestational age at the time of screening in both screening groups.

#### Secondary outcome 3: time to access antenatal diabetes care for women with GDM

The time to access GDM treatment for positive GDM cases in the primary and secondary care settings is a secondary outcome in the RCT. The Diabetes Clinical Management System (DIAMOND) will be used to collect data on the date of first access to specialist diabetes antenatal care for women diagnosed with GDM. This will enable analysis of time of access to care in both screening groups.

#### Secondary outcome 4: pregnancy outcomes for women with GDM

Pregnancy outcomes for positive GDM cases in the primary and secondary care settings are a secondary outcome in the RCT. The DIAMOND Diabetes Clinical Management System will be used for the collection of data on a range of maternal and neonatal outcomes for women diagnosed with GDM (Table 2).

**Table 2:** Primary and secondary outcomes, and assessment method

**Primary/ secondary**	**Outcome variable**	**Assessment method**
Primary	Uptake of screening	laboratory database
Secondary 1	GDM prevalence	laboratory database
Secondary 2	Timing of screening	laboratory database
Secondary 3	Time to access antenatal diabetes care	Hospital database
Secondary 4	Pregnancy outcomes:	Hospital database
	Maternal outcomes	
	Caesarean section delivery	
	Assisted normal delivery	
	Hypertension	
	Pre-eclampsia texaemia (PET)	
	Antepartum haemorrhage (APH)	
	Post-partum haemorrhage (PPH)	
	Neonatal outcomes:	
	Miscarriage	
	Foetal death intrauterine (FDIU)	
	Stillbirth	
	Admission to neonatal intensive care unit (NICU)	
	Length of stay at NICU	
	Gestational age	
	Size for gestational age	
	Congenital malformations	
	Apgar scores	
	Respiratory distress	
	Hypoglycemia	
	Jaundice	
	Composite perinatal score (neonatal hypoglycemia, respiratory distress, need for phototherapy, birth trauma, 5-minute Apgar score less than 7, or prematurity).	

### Analysis plan

Differences in patient characteristics in primary care versus secondary care will be presented using suitable numerical and graphical summaries. A nonlinear mixed model for a binomial response will be used to compare the proportion of uptake between those in primary and secondary care while adjusting for differences in patient characteristics at baseline as appropriate. The analysis of the secondary responses will involve linear and nonlinear mixed models for continuous and categorical responses and survival models for the analysis of time to event data. The main analysis will follow an intention-to-treat strategy; sensitivity analyses and per protocol analysis will be used to explore the effect of departures from the assumption made in the main analysis. All analyses will be performed using R Statistical advice and analysis will be provided by Dr. John Newell of the HRB Clinical Research Facility, NUI Galway.

### Study management

A study management group will be formed comprising the principal investigator, co-investigators and trial statistician. A data monitoring committee is not required as the study management group will liaise every month to review interim analysis and monitor adherence. The principal investigator will make the final decision to terminate the trial in the event that uptake rates fall below pre-trial levels in either intervention arm. It is not anticipated that there will be premature termination of study. However, if this occurs, then the data will be analysed and results circulated to team members. The ethics committee will be notified of premature termination and the reasons for termination. Adverse events or other unintended effects of trial interventions that come to the attention of trial investigators will be reported immediately to the study management Group. All protocol amendments will be submitted to the ethics committee for review and approval before implementation and to trial registries.

### Data protection and participant confidentiality

Data acquired according to this protocol will be recorded on a password-protected database on a secure network. All entries will be made by an authorised member of the investigator’s staff. Data will be entered into the study database and verified through the use of programmed edit checks for accuracy and completeness. The corrected data and a complete audit trail of corrections will be retained. An internal audit of a sample of the data will be conducted quarterly to assure quality.

The investigator will insure that participants’ anonymity is maintained. Participants will not be identified by their names, but by their assigned identification number and initials. The investigator will keep a separate log of subjects’ identification numbers, names, addresses, telephone numbers and hospital numbers. Signed informed consent forms, will be maintained securely. Only authorized members of the investigator’s staff will have access to the final trial dataset.

All information regarding the study data or results supplied to the investigator is privileged and confidential information. The investigator agrees to use this information to accomplish the study and will not use it for other purposes.

### Economic evaluation

The economic evaluation will incorporate both cost-effectiveness analysis and cost-utility analysis for the alternative screening options. The basic tasks of the evaluation are to identify, measure, value and compare the costs and outcomes of the alternative screening locations. A societal-costing perspective will be adopted as costs are likely to fall on patients, their families and the state. The healthcare resources consumed will reflect the costs of organising and operating the two programmes. These costs will reflect the time input of health professionals and fixed or overhead costs. All contacts with the health services will be recorded and valued. The patient and family costs include any out-of-pocket expenses and any own time input into the screening process, including time foregone as part of any treatment process. To complete the analysis, cost data will be combined with effectiveness data on the primary outcome from the trial; that is, uptake of screening. In addition, Quality Adjusted Life-Years (QALYs) will be estimated in order to enable comparisons with interventions from within and beyond maternity care. To this end, modeling techniques to compute QALYs will be employed. The analysis will use standard economic analysis for the calculation of costs and incremental cost-effectiveness ratios. Unit costs will be applied to the resource use data to calculate the various costs of care. Comparisons between the two screening groups will be made on the outcomes above and costs will be assigned accordingly. Analyses will report the incremental cost effectiveness from a societal perspective and from a publicly funded health system perspective in line with Health Information Quality Assessment (HIQA) guidance [36]. Budget-impact analyses for the primary and secondary care based screening strategies will be undertaken. Uni- and multivariate sensitivity analyses, as well as a probabilistic sensitivity analysis, will be used to address uncertainty in the study.

### Qualitative analysis

#### Pre-trial phase

In order to maximize user involvement in the design of the screening process, pre-trial interviews and focus groups will be held with users and stakeholders including women with previous experience of GDM screening, primary care teams both urban and rural, and secondary care teams.

#### Post-trial phase

In order to explore the psychological impact of GDM screening and diagnosis on maternal experience and well-being, post-trial interviews and focus groups will be held with women from both the primary care and secondary care screening groups, and women who tested positive and negative for GDM at each site. Attitudes to and perceptions of GDM screening in primary and secondary care, including barriers and facilitators of screening at each site, will also be explored. In addition, the attitudes and experiences of stakeholders will be explored through interviews and focus groups with stakeholders involved in the trial. Stakeholder groups will include GP practice staff, hospital obstetric outpatient staff, and laboratory staff.

The findings of the qualitative analysis, along with the findings from the trial itself, will inform the recommendations for a national GDM screening programme.

## Trial status

Enrolment into the study will begin in January 2013. Recruitment is expected to be complete by December 2014.

Ethical approval for this research was obtained from the Clinical Research Ethics Committee, Galway University Hospitals, Health Service Executive of Ireland, 5 December 2012 (Ref: C.A.753).

## Endnotes

^a^The Atlantic DIP Network was established in 2005 to provide robust information on pregnancy outcomes for women with diabetes across five regional hospital centres along the Irish Atlantic seaboard. ^b^Medical Card status refers to a card issued by the Health Service Executive which entitles the holder of the card to a range of Health Services free of charge.

## Abbreviations

APH: Antepartum haemorrhage; Atlantic DIP: Atlantic diabetes in pregnancy; DIAMOND: The diabetes clinical management system; FDIU: Foetal death intrauterine; GDM: Gestational diabetes mellitus; GP: General practitioner; GUH: Galway University Hospital; HRB: Health Research Board; IADPSG: International Association of Diabetes and Pregnancy Study Groups; MGH: Mayo General Hospital; NICU: Neonatal intensive care unit; OGTT: Oral glucose tolerance test; PET: Pre-eclampsia toxaemia; PPH: Post-partum haemorrhage; QALY: Quality-adjusted life year; RCT: Randomised controlled trial; WHO: World Health Organisation.

## Competing interests

The authors declare that they have no competing interests.

## Authors’ contributions

AOD and JI are involved in trial management, recruitment, and acquisition of baseline data; SF and OT are involved in recruitment and acquisition of baseline data. AOD drafted the manuscript. PG, LG, BM and FD conceived and designed the study; PG will also carry out the economic analyses. JN participated in the design of the study, performed the sample size and power calculations, and developed the plan for statistical analysis of trial outcomes. FD revised the manuscript, and gave final approval of the version to be published. All authors read and approved the final manuscript.

## Authors’ information

AOD is a research psychologist currently working as a post-doctoral researcher in medicine. JI is a social anthropologist currently working as a post-doctoral researcher in medicine. OT and SF are medical students at the School of Medicine at the National University of Ireland Galway (NUI Galway). PG is a health economist and lecturer in the J.E. Cairnes School of Business & Economics at NUI Galway. LG is a GP in practice in County Clare, Ireland. LG is also founding clinical director of the Western General Practice Research and Education Network in Ireland and senior lecturer in General Practice at NUI Galway. BM is a senior lecturer in clinical psychology, director of the Doctor of Psychological Science programme in Clinical Psychology at NUI Galway, and joint director of the Centre for Pain Research. JN is a senior lecturer in biostatistics in the Clinical Research Facility at NUI Galway. FD is a consultant endocrinologist at the University Hospital Galway; and lead principal investigator of this study, responsible for the scientific and technical direction of the research programme.
